# Karyopherin Alpha Proteins Regulate Oligodendrocyte Differentiation

**DOI:** 10.1371/journal.pone.0170477

**Published:** 2017-01-20

**Authors:** Benjamin M. Laitman, John N. Mariani, Chi Zhang, Setsu Sawai, Gareth R. John

**Affiliations:** 1 Friedman Brain Institute, New York, New York, United States of America; 2 Corinne Goldsmith Dickinson Center for Multiple Sclerosis, New York, New York, United States of America; 3 Neurology, Icahn School of Medicine at Mount Sinai, New York, NY, New York, United States of America; Instituto Cajal-CSIC, SPAIN

## Abstract

Proper regulation of the coordinated transcriptional program that drives oligodendrocyte (OL) differentiation is essential for central nervous system myelin formation and repair. Nuclear import, mediated in part by a group of karyopherin alpha (Kpna) proteins, regulates transcription factor access to the genome. Understanding how canonical nuclear import functions to control genomic access in OL differentiation may aid in the creation of novel therapeutics to stimulate myelination and remyelination. Here, we show that members of the Kpna family regulate OL differentiation, and may play distinct roles downstream of different pro-myelinating stimuli. Multiple family members are expressed in OLs, and their pharmacologic inactivation dose-dependently decreases the rate of differentiation. Additionally, upon differentiation, the three major Kpna subtypes (P/α2, Q/α3, S/α1) display differential responses to the pro-myelinating cues T3 and CNTF. Most notably, the Q/α3 karyopherin *Kpna4* is strongly upregulated by CNTF treatment both compared with T3 treatment and other Kpna responses. *Kpna4* inactivation results in inhibition of CNTF-induced OL differentiation, in the absence of changes in proliferation or viability. Collectively, these findings suggest that canonical nuclear import is an integral component of OL differentiation, and that specific Kpnas may serve vital and distinct functions downstream of different pro-myelinating cues.

## Introduction

Precise transcriptional regulation of oligodendrocyte (OL) differentiation, influenced by extrinsic stimuli, is required for OL development and subsequent central nervous system (CNS) myelination [1]. As myelin is essential for efficient action potential transmission in the CNS [2], failure of myelination or loss of myelin (demyelination) results in symptoms of white matter diseases such as leukodystrophies and multiple sclerosis (MS) [3,4]. Myelin repair (remyelination) restores conduction and brings functional recovery in diseases such as MS, but is inefficient, and often fails in the disease’s later stages [1]. Disruption of white matter formation and repair is characterized by failure of oligodendrocyte progenitors (OLPs) to mature into myelinating OL [1]. Currently, treatments to promote OL differentiation, and thus myelin formation and myelin repair, do not exist. A better understanding of transcriptional control of OL differentiation, including access to the genome, will aid in the generation of novel therapeutics to stimulate myelination and remyelination, bringing a return of function to patients.

**Fig 1:**
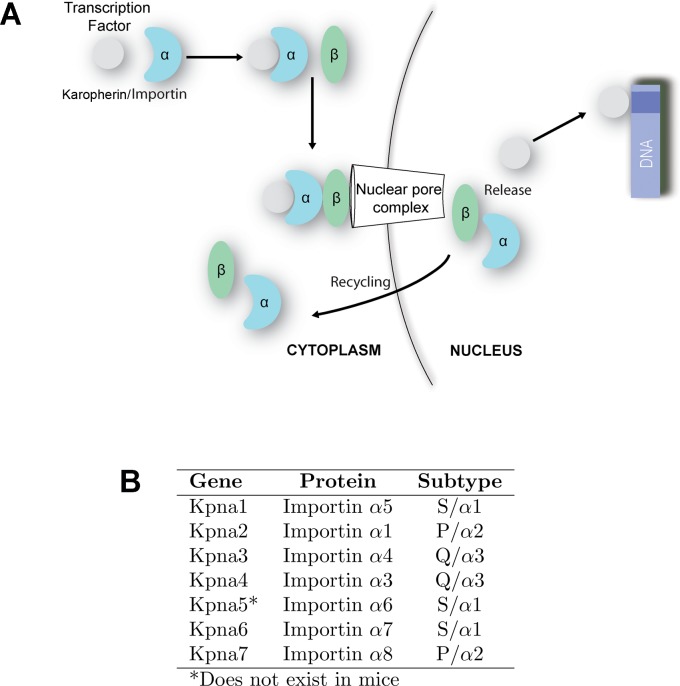
Karyopherin alphas in canonical nuclear import. **(A)** In canonical import, a karyopherin α (Kpna) binds to both the nuclear localization sequence-containing cargo protein and to Kpnb. Subsequently, Kpnb interacts with the nuclear pore to bring the trimeric complex into the nucleus. Once within the nucleus, the complex dissociates, and Kpna and Kpnb are recycled to the cytoplasm. **(B)** Seven *Kpna* genes encoding Kpna proteins have been identified in humans, and six in mice. They belong to three subtypes based on homology. Nomenclature for each corresponding Impα protein is indicated. While nomenclature of the paralogs varies in different studies, we will use the human Kpna designations for clarity.

Proper tissue functioning depends on coordinated spatio-temporal gene expression, which is impacted by efficient nuclear import of transcription factors and epigenetic modifiers. Larger cargo requires active transport by karyophillic import proteins (known as karyopherins or importins) [5]. Here, for clarity we will use the karyopherin (Kpna/Kpnb) nomenclature preferentially throughout the text. In the canonical nuclear import program, a heterodimer of karyophern alpha (Kpna) and beta (Kpnb) targets proteins to the nucleus and is responsible for the transport of hundreds of factors through the nuclear envelope (**Fig 1A**) [6,7]. During this process, a Kpna binds to both the nuclear localization sequence-containing cargo and to Kpnb. Subsequently, Kpnb interacts with the nuclear pore to bring the trimeric complex into the nucleus. Multiple Kpna subtypes exist (**Fig 1B**), and recent evidence from other lineages has demonstrated that they have different cargo specificities and stage-specific expression during differentiation [8–13]. Thus, one mechanism to modulate gene expression in OL during development and remyelination may rely on the existence of different paralogs of nuclear import proteins controlling which crucial transcription factors enter the nucleus and when.

While Kpna and Kpnb are expressed in the CNS with a distribution of different isoforms in varied regions [14–16], less is known about karyopherin expression, regulation, and nuclear import in the OL lineage. In contrast, studies have identified important roles for nuclear export of differentiation inhibitors, such as p57kip2 [17] or acetylated-Olig1 [18], prior to the onset of OL maturation. The few examinations of karyopherins in OL have focused on Kpnb in pathologic contexts [19–21]. Chronic seizures, for example, result in increased expression of Kpnb in hippocampal NG2^+^ glial cells (polydendrocytes) [21]. Conversely, in demyelinating diseases such as MS, Kpnb functional activity, and thus nuclear import, may be reduced. Microarray data from human MS lesions, for example, reveals decreased expression of Kpnb [22], and other studies have reported that the negative regulator of OL differentiation 30kDa HIV-1 Tat interacting protein (TIP30) inhibits Kpnb mediated nuclear import of the Notch intracellular domain (NICD) and Olig1 [19,20,23]. As Kpnb is central to the import process, it is logical to examine its role in these contexts. However, since there are a variety of distinct Kpna paralogs, studying their roles in development and disease may elucidate a set of novel mechanisms regulating gene expression in OLs. Indeed, we recently identified Kpna1 as an important regulator of OL differentiation, inactivation of which disrupts OL maturation and impacts viability [24]. In our recent study, we did not explore potential roles of other Kpna family members in OL development. However, the involvement of Kpna1 in the differentiation program, and the tendency of multiple Kpna paralogs to play critical roles in maturation processes, suggests that other family members may fulfill as yet undiscovered functions in OL differentiation.

Here, to determine the extent to which different Kpna isoforms perform physiologic roles in OL differentiation, we have profiled karyopherin expression patterns in specified OLP and during differentiation in response to two different extrinsic pro-myelinating cues, the gp130 receptor ligand ciliary neurotrophic factor (CNTF) [25] and the thyroid hormone tri-iodothryronine (T3) [26,27]. Our data demonstrate expression of five Kpna paralogs in these cells, and that Kpnas display differential responses to pro-myelinating factors during differentiation. Compatible with our previous data, we also show that nuclear import is an important mechanism regulating OL differentiation and viability. Following these discovery steps, we more closely studied one karyopherin alpha paralog, Kpna4, which we have found to be highly CNTF-sensitive and for a regulator of differentiation. To our knowledge, this is the first direct examination of Impα expression profiles in OL. Results from this study provide a fuller understanding of this canonical pathway in development, and may illuminate unique avenues to control the transcriptional program in an effort to promote myelin formation and repair in patients with white matter disease.

## Results

### Karyopherin alpha isoforms are differentially expressed in oligodendrocyte progenitor cells

**Fig 2:**
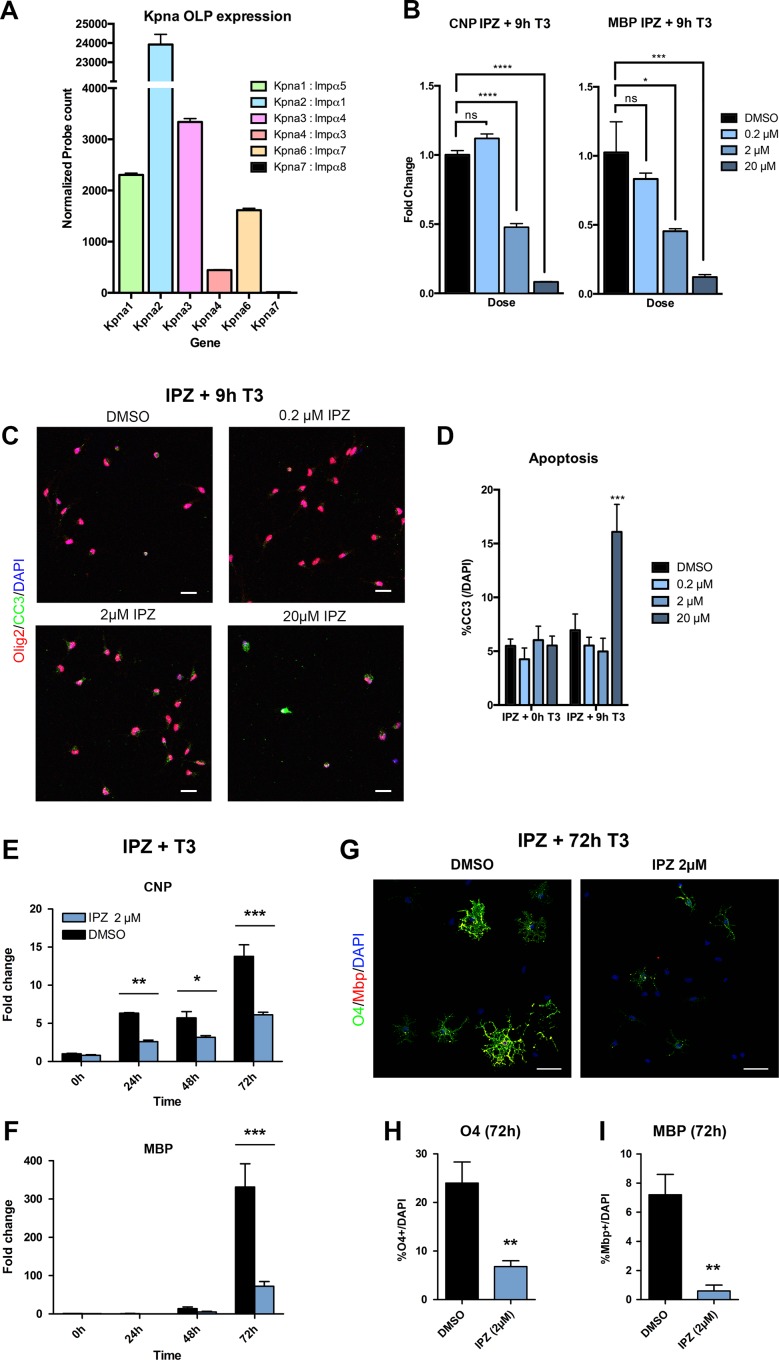
Karyopherin alphas are widely expression in OLP and important regulators of OL differentiation. **(A)** *Kpna* gene expression in OLP exposed only to proliferative factors (PDGFAA, 10 ng/ml and bFGF, 20 ng/ml) for 24h. Expression was quantified from isolated total RNA with NanoString nCounter Gene Expression Assay. A panel of probes was designed for target genes and normalized to housekeeping genes *Alas1*, *Ppia*, *Gapdh*, *Actb*, and *Rps11*. Following assay completion, raw data was normalized and analyzed using nSolverTM software. *Kpna2* is the highest expressing Impα in OLP. *Kpna7* is not expressed in oligodendrocytes. **(B-D)** Results from primary OLP pre-treated 1h with increasing concentrations the karyopherin inhibitor importazole (IPZ) or DMSO, then differentiated with T3 (40ng/ml) in the presence of IPZ/DMSO for 9h and harvested for qPCR **(B)** or immunocytochemistry **(C)**. **(B)** Failure of karyopherin functioning in IPZ-treated cultures results in dose-dependent inhibition of differentiation as measured by a decrease in the expression of the OL maturation markers *CNP* or *MBP* in qPCR. **(C,D)** While, at lower doses of IPZ, inhibition of differentiation is seen in the absence of any impact on viability, higher concentrations of IPZ eventually produce apoptosis, quantified as the percentage of DAPI cells that are cleaved-caspse 3 (CC3) positive. **(E-I)** Extension of differentiation out to 72h with T3 in the presence of 2μM IPZ confirms this reduction in maturation. Results are from primary OLP pre-treated 1h with 2μM IPZ or DMSO, then differentiated with T3 (40ng/ml) in the presence of IPZ/DMSO for up to 72h and harvested for qPCR **(E,F)** or immunocytochemistry **(G-I)**. **(E,F)** Failure of karyopherin functioning in 2μM IPZ-treated cultures results in inhibition of differentiation as measured by a decrease in the expression of the OL maturation markers *CNP* or *MBP* in qPCR. **(G)** Representative image shows maturation markers for immature/mature OL (O4), and mature OL (MBP) in the OL lineage in IPZ-treated cultures and DMSO controls treated with T3 for 72h. Maturation was assessed by quantifying %O4/DAPI **(H)** and %MBP/DAPI **(I)** expressing cells. Data presented are mean ± S.E.M. and representative of 3 **(A,B,C,E,F)** or 5 **(H,I)** independent cultures. Statistics, **(B)** One-way ANOVA plus Bonferroni post-test, **(D,E,F)** Two-way ANOVA plus Bonferroni post-test, **(H,I)** Student’s t-test, *p<0.05, **p<0.01, ***p<0.001, ****p<0.0001. Scale bars, **(C,G)** 20μm. Individual data values are in **S1 Data**.

Our first step in examining the roles of Kpna paralogs in OL development was to determine which are expressed in the lineage, using NanoString analysis of primary mouse OLP cultures. In accordance with an independently published transcriptome dataset [28], we found that *Kpna1*, *2*, *3*, *4*, and *6* all exhibited detectable normalized probe counts above NanoString negative control counts, while *Kpna7* did not (**Fig 2A** and **S1 Table**). *Kpna2* overwhelmingly displayed the highest expression among Impαs, with an average probe count of 23,927.0 ± 527.5 a.u., compared to the average count of the other Impαs in OLP, 1,925.0 ± 318.1 a.u. (p<0.0001; mean ± S.E.M.; a.u.: arbitrary units). The remaining members displayed lower levels of expression (from highest to lowest): 3,339 ± 65.6 (*Kpna3*), 2,304.0 ± 35.5 (*Kpna1*), 1,614 ± 38.4 (*Kpna6*), and 442.2 ± 11.0 (*Kpna4*) (for frame of reference, expression of housekeeping control transcripts *Gapdh* and *Actin* was 70,077 ± 397.2 and 153,795 ± 2222.0 a.u. respectively). While NanoString analysis is impacted by probe binding efficiency, these data do suggest that *Kpna2* displays significantly higher expression than other paralogs in specified OLP.

### Canonical transport is an important regulatory mechanism in oligodendrocyte differentiation

With widespread expression detected, we hypothesized that canonical import was essential to OL development, particularly during differentiation, a period when a wide range of transcription factors undergo coordinated entry into the nucleus. To test this hypothesis, we differentiated mouse OLP using T3 in the presence of increasing concentrations of importazole (IPZ), a well-characterized pharmacological inhibitor of Kpna/b-mediated nuclear import [29]. Importantly, inactivation of all canonical import strongly inhibited OLP differentiation as evidenced by a dose-dependent inhibition of the normal increase in expression of maturation and myelin markers (*CNP* and *MBP*) detected via quantitative PCR (qPCR) (**Fig 2B**). Moreover, although concentrations of IPZ up to 2μM produced dose-dependent inhibition of differentiation in the absence of any impact on viability, the highest dose of IPZ used (20μM) produced an increase in apoptosis, observed as a rise in the percentage of cleaved-caspase 3 (CC3) expressing cells (**Fig 2C and 2D**). Thus, while the effects of decreased expression of maturation markers at the highest dose of IPZ may relate in part to the loss of cells due to programmed death, lower doses produced reductions in maturation marker expression without changes in viability (**Fig 2B**). Indeed, extension of differentiation out to 72h with T3 in the presence of 2μM IPZ confirmed a sustained reduction in maturation (**Fig 2E–2I**). As such, these results suggest that canonical nuclear import is an important regulatory mechanism in OL differentiation, and may also play a broader role in viability.

#### Karyopherin alphas exhibit differential transcriptional profiles during oligodendrocyte differentiation

Since Kpna/b-mediated nuclear import regulates OL differentiation, we wondered how expression of each paralog changes in the differentiation program. During differentiation in other lineages, a phenomenon known as isotype switching has been observed, whereby Kpna isoforms swap expression predominance in order to enact a switch from maintenance of the progenitor state towards progression of differentiation [11,12]. However, this is not a universal mechanism used by all cells [13,30]. Given the high expression in OLP of one alpha isoform (*Kpna2*) compared to the rest, we hypothesized that the potential for differential changes in Kpna isotype expression may exist in these cells. Additionally, we suspected that different isotypes would be selectively altered based on the pro-myelinating cue used, since different Kpnas could carry a different set of transcription factors (i.e. different cargo) as part of downstream mechanisms specific to each cue.

**Fig 3:**
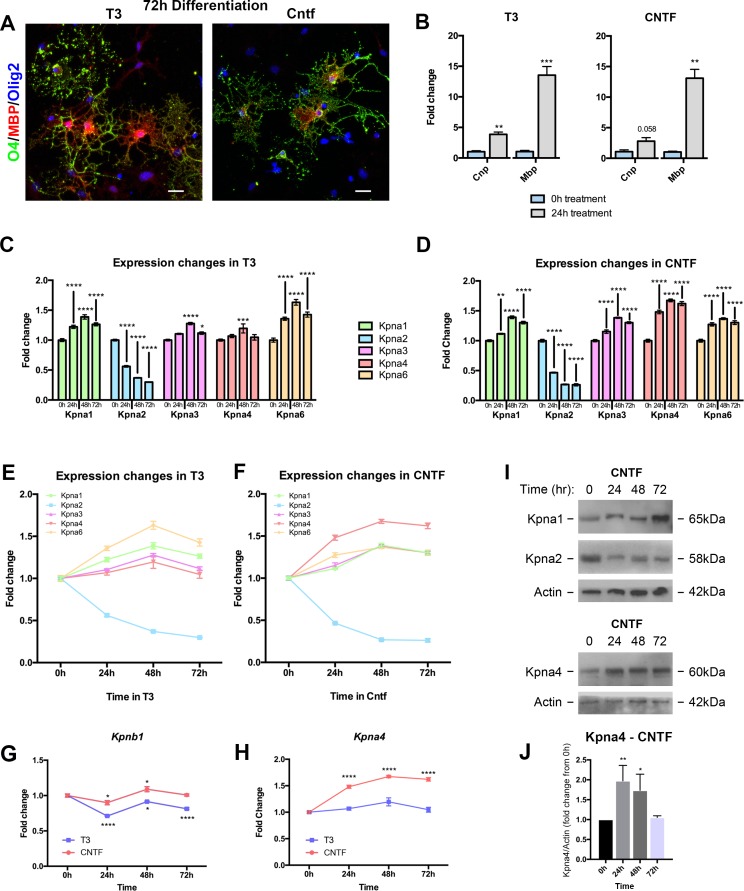
Oligodendrocyte Kpna expression alters with differentiation. **(A,B)** T3 and CNTF both induce differentiation of OL. **(A)** Confocal images of mature OL treated with either T3 (60ng/ml) or CNTF (100ng/ml) for 72h. Cells were immunostained for the earlier maturation marker, O4 (green), and the later myelin protein, MBP (red), a marker of mature OL. **(B)** Results from qPCR of fold change expression for the maturation markers *CNP* and *MBP* at 24h following T3 (left) or CNTF (right) treatment, compared to 0h. **(C-H)** *Kpna* gene expression data from OL lineage for 72h treatment with either T3 or CNTF, analyzed at 24h intervals. Transcripts were quantified from isolated total RNA using NanoString nCounter Gene Expression Assay. A commercially available panel of probes for target genes was normalized to housekeeping genes *Alas1*, *Ppia*, *Gapdh*, *Actb*, and *Rps11*. Following assay completion, raw data was analyzed using nSolver software before being subjected to statistical analysis. **(C-F)** Expression fold change in response to T3 (60ng/ml) or CNTF (100ng/ml) at 24h intervals, derived from NanoString analysis. Results demonstrate differential changes in expression of Kpna isotypes during differentiation. *Kpna2* expression strongly decreases, whereas all other isoforms increase. The three Kpna subtypes (P/α2, Q/α3 and S/α1) also respond differently to the extrinsic factor used. While *Kpna2* (Subtype P/α2) decreases no matter the cue, *Kpna4* (Subtype Q/α3) shows greater changes in expression in response to CNTF, and *Kpna1* and *Kpna6* (Subtype S/α1) display greater fold changes in response to T3 than other subtypes, in addition to responsiveness to CNTF. **(G)** Expression fold change derived from NanoString analysis shows that *Kpnb1* displays relatively stable expression throughout the course of OL differentiation. **(H)** Expression fold change derived from NanoString analysis demonstrates differential responses of *Kpna4* to CNTF versus T3 treatment. While expression increases slightly in response to T3, the response to CNTF is greater at all time points beyond 0h. **(I,J)** Gene expression changes resulted in corresponding alterations in protein levels in response to CNTF. OLP treated for up to 72h with CNTF (100ng/ml) were harvested and immunoblotted for Kpna1, Kpna2, and Kpna4, with Actin used as a loading control **(I)**. **(J)** Accompanying densitometry plots for Kpna4 were calculated from the ratio of Kpna4/Actin pixel intensity and displayed as fold change from 0h of CNTF treatment. Data are mean ± S.E.M. and representative of 3 independent cultures. Statistics, **(B)** Student’s t-test, **(C-H)** Two-way ANOVA plus Bonferroni post-test, **(J)** One-way ANOVA plus Bonferroni post-test, *p<0.05, **p<0.01, ***p<0.001, ****p<0.0001. Statistics for **(E-F)** are in **S2 Table**. Scalebar: **(A)** 20μm. Individual data values for **(C-H)** are in **S1 Table** and **S1 Data**. Individual data values for **(B,J)** are in **S1 data**.

To test this hypothesis, we differentiated OLP using either T3 (60 ng/ml) or CNTF (100 ng/ml) and harvested for RNA at 24h intervals through 72h. This latter time-point corresponds to the emergence of mature and arborized OL positive for the early maturation marker, O4, and the later myelin protein, MBP regardless of the differentiating factor used (**Fig 3A**). Equivalent maturation is also observed at earlier time points, as demonstrated by qPCR confirming similar increases in RNA for the myelin components *CNP* and *MBP* in response to T3 and CNTF by 24h of differentiation (**Fig 3B**). Kpna paralog expression was thus assessed from 0-72h of treatment with T3 or CNTF, at 24h intervals.

Notably, while the ranking of expression levels in OLP was maintained throughout differentiation and seen in more mature OL at 72h (see **S1 Table**), these studies showed that each individual Kpna underwent significant changes in expression during the differentiation program. Differential changes in expression of Kpna paralogs were detectable from 24h of differentiation (**Fig 3C–3F**). We found that *Kpna2* decreased by approximately 70% of its OLP value in response to both T3 and CNTF. In contrast, the other paralogs all increased their expression during differentiation regardless of the initiating stimulus, though with different magnitudes (**Fig 3C and 3D**). Thus, the predominant response of Kpnas is to increase expression of family members with differentiation. We compared these results to *Kpnb1*, the binding partner for Kpna within the Kpna/b heterodimer. Unlike the significant increases seen in the expression of Impαs, *Kpnb1* showed more stable levels throughout differentiation, albeit with slight fluctuations in expression compared to its OLP levels (**Fig 3G**).

Intriguingly, these data further revealed that Kpna subtypes respond differently to different pro-myelinating cues, as evidenced by differential responses to T3 versus CNTF. While *Kpna2* (Subtype P/α2) decreased no matter the extrinsic factor, *Kpna4* (Subtype Q/α3) showed a greater response to CNTF compared to other members (**Fig 3C–3F and 3H, S1 Table**). Conversely, *Kpna1* and *Kpna6* (Subtype S/α1) displayed greater fold changes in response to T3 compared to members of subtypes P/α2 and Q/α3, as well as responsiveness to CNTF (**Fig 3F and S1 Table**). These findings were confirmed via immunoblotting for representative family members (Kpna1, Kpna2, and Kpna4) in response to CNTF (**Fig 3I and 3J**). Thus, each of the three major Kpna subtypes (P/α2, Q/α3, S/α1) showed a differential response to T3 and CNTF: P/α2 decreased expression to both, Q/α3 increased more substantially to CNTF, and S/α1 had greater responsiveness to T3 than other subtypes.

### Effects of inactivation of *Kpna4* is more severe in the presence of CNTF differentiation

**Fig 4:**
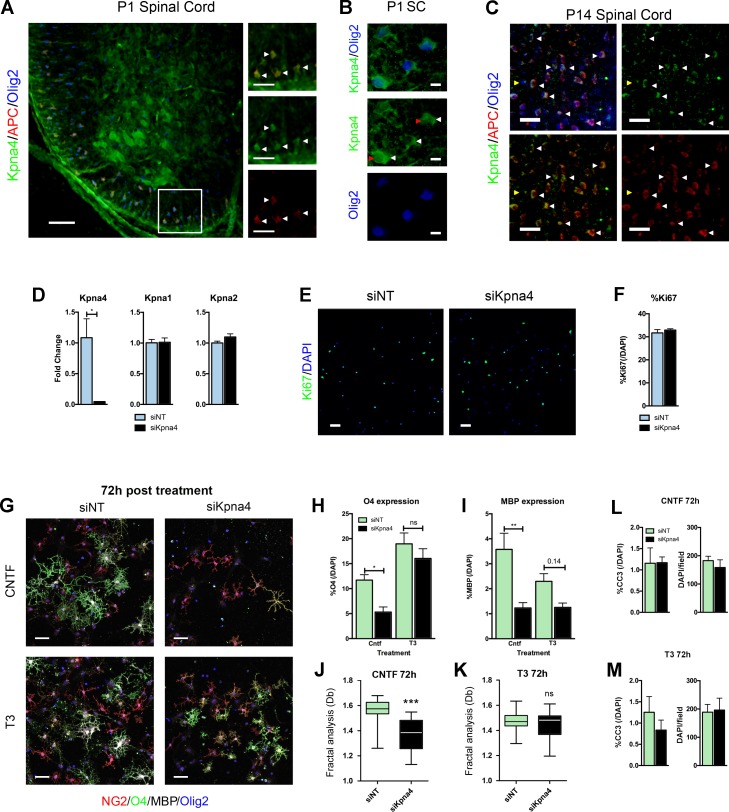
*Kpna4* is an important regulator of CNTF-induced oligodendrocyte differentiation. **(A-C)** Confocal imaging of Kpna4 expression *in vivo* in P1 **(A)** and P14 **(C)** thoracolumbar mouse spinal cord sections. Kpna4 is expressed in multiple cells within the CNS including APC^+^Olig2^+^ OLs (representative cells indicated with white arrows), but not by immature Olig2^+^APC^-^ cells (yellow arrowhead). The region outlined in **(A)** is magnified in panels to the right. Panel **(B)** shows a high power image of Olig2^+^ cells in thoracolumbar spinal cord at P1. Cells show both nuclear (**B,** red arrowheads) and cytoplasmic Kpna4 immunoreactivity (**B,** white arrowheads). **(D)** qPCR data from OLP nucleofected with *siKpna4* or *siNT* control for 24h. Silencing is efficient and is selective for *Kpna4*. **(E,F)** Representative confocal image and associated quantification from primary mouse OLP silenced for *Kpna4* versus nontargeting control, then allowed to proliferate in the presence of the mitogens PDGFAA and bFGF for 24h. Active proliferation was assessed by immunocytochemistry for Ki67, confocal imaging, and quantitation of %Ki67/DAPI cells. **(G-I)** To directly compare responses of Kpna4-deficient cells and controls to CNTF vs. T3 in parallel, primary cultures were nucleofected with siRNA for *Kpna4* or nontargeting (NT) control, then treated with either CNTF or T3. **(G)** Representative confocal image and associated quantification from primary mouse OLP nucleofected with *siKpna4* or *siNT* control and then differentiated with T3 (60 ng/ml) or CNTF (100 ng/ml) for 72h. This image shows maturation markers for OLP (NG2), immature/mature OL (O4), and mature OL (MBP) in the Olig2^+^ OL lineage in *Kpna4*-deficient cultures and controls treated with T3 or CNTF. Maturation was assessed by quantifying %O4/DAPI **(H)** and %MBP/DAPI **(I)** expressing cells. Maturation was reduced in CNTF-treated cultures. In T3-treated cultures, only a slight reduction was observed in the proportion of MBP expressing cells, which did not reach significance, illustrating a differential impact of silencing *Kpna4* depending on the growth factor added to induce differentiation. **(J,K)** The reduction in maturation markers coincided with a loss in complexity of branching morphology, a marker of OL maturity, as assessed by fractal analysis. Fractal results are represented by the box-counting fractal dimension (Db). **(L,M)** Cell death was unchanged in these cultures regardless of treatment with CNTF **(L)** or T3 **(M)**, measured by both assessments of apoptosis (%Cleaved-caspase 3 (CC3)/DAPI) and total cell number (DAPI counts per field). Data are mean ± S.E.M. and representative of 3 **(D,F,L,M)** or 5 **(H-K)** independent cultures. Statistics, **(D,F,L,M,J,K)** Student’s t-test, **(H,I**) Two-way ANOVA plus Bonferroni test, *p<0.05, **p<0.01, ***p<0.001. Scalebars, **(A)** 50μm, 25μm inset, **(B)** 10μM, **(C)** 25μm, **(E)** 20μm, **(G)** 20μm. Individual data values are in **S1 data**.

Since we have previously shown that *Kpna1* is an important regulator of OL differentiation, our attention was drawn to the data for *Kpna4* in the above studies (**Fig 3H–3J**), as it showed the most striking difference in response to pro-myelinating cues, as well as one of the greatest magnitude changes associated with differentiation, which additionally was CNTF-specific. To determine *in vivo* relevance of our NanoString findings for *Kpna4*, we assessed its expression in developing spinal cord, a well-characterized system for studying myelination *in vivo* [1]. In the spinal cord myelination proceeds with a rostral to caudal gradient (especially ventrally) [31]. Conversely in the brain it progresses from caudal sites to rostral ones. CNS myelination is extensive by postnatal day 14 (P14). Confocal imaging of P1 and P14 thoracolumbar spinal cords demonstrated widespread immunoreactivity for Kpna4 in OL expressing the lineage marker Olig2 and the maturation marker APC in the more mature ventrolateral funiculi, but not by immature Olig2^+^APC^-^ cells (**Fig 4A and 4C**). These cells exhibited both nuclear and cytoplasmic Kpna4 expression, compatible with its function in shuttling cargo between both regions (**Fig 4B**).

Interestingly, *Kpna4* inactivation in primary cultures implicated this import factor as an important regulator of the OL differentiation program downstream of CNTF-induced signaling. To examine its involvement, we inactivated *Kpna4* via nucleofection of an siRNA targeted specifically to *Kpna4* and compared it to a nontargeting (*siNT*) control. To directly compare responses of Kpna4-deficient cells and controls to CNTF vs. T3 in parallel, primary cultures were nucleofected with siRNA for *Kpna4* or nontargeting (NT) control, then treated with either CNTF or T3. *Kpna4* inactivation was efficient and selective—other isoforms did not decrease with silencing (**Fig 4D**). *Kpna4* silencing did not alter active proliferation of OLPs as assessed by immunocytochemistry for Ki67 followed by confocal imaging and quantitation of %Ki67^+^ (%Ki67/DAPI) cells. The percentage of Ki67^+^ OLP was unchanged between groups in primary cultures (**Fig 4E and 4F**). Following exposure of OLP to either T3 or CNTF, using confocal microscopy we determined the percentage of OL expressing the maturation markers O4 and MBP. Compatible with its expression patterns in our NanoString analysis (see **Fig 3**), differentiation was strongly inhibited by *Kpna4* inactivation in CNTF-treated cultures (**Fig 4G–4K**). There was a strong reduction in the accumulation of O4-expressing cells in CNTF-treated cultures deficient in *Kpna4* versus nontargeting controls (**Fig 4H**). Similarly, the induction of MBP expression was also substantially decreased in CNTF-treated cultures in which *Kpna4* was silenced (**Fig 4I**). Cells also displayed a less complex branching morphology, as measured by fractal analysis (**Fig 4J**). Conversely, we observed only a slight trending decrease in the percentage of MBP expressing cells in T3-treated cultures in which *Kpna4* was inactivated (p = 0.14) (**Fig 4I**), with no significant difference in arborization (**Fig 4K**), in line with the more minor increase in *Kpna4* expression observed in response to T3 treatment. This suggests that *Kpna4* inactivation may slightly delay differentiation to the more mature stage in response to T3, whereas in CNTF-treated cultures its loss causes a more severe inhibition or delay of differentiation. Importantly, these changes seen in differentiation were not due to cell death, as both cell number and viability (measured as the percentage of apoptotic cells displaying cleaved-caspase 3) remained unaltered after *Kpna4* inactivation, compared to *siNT* control, in both T3- and CNTF-treated cultures (**Fig 4L and 4M**).

## Discussion

This study shows that Kpnas are important regulators of OL differentiation, and suggests that different members of the family may play distinct roles downstream of different pro-myelinating stimuli. In particular, widespread Kpna expression is present in and regulates OL differentiation, and OLP display differential changes in Kpna isotype expression from the onset of differentiation. In addition, the three major Kpna subtypes (P/α2, Q/α3, S/α1) show differential responses to different pro-myelinating factors (in our experiments, T3 and CNTF). Subtype P/α2, epitomized by *Kpna2*, decreases in expression following T3 and CNTF treatment, Q/α3 factors (*Kpna4* and *Kpna3*) increase substantially in response to CNTF, and S/α1 members (*Kpna1* and *Kpna6*) display responsiveness to T3 as well as to CNTF. Interestingly, while we have previously reported CNTF-mediated regulation of Kpna1 [22], our findings here identify additional sensitivity to T3 in primary cultures. Finally, loss of the highly CNTF-responsive *Kpna4* results in inhibition of CNTF-induced differentiation, in the absence of changes in proliferation or viability. Collectively, these findings suggest that canonical nuclear import is an integral part of OL differentiation, and that specific Kpna paralogs may fulfill important functions downstream of pro-myelinating cues. The array of nuclear import proteins expressed in the OL lineage may relate to the presence of diverse cargo specificities [9] and to potentially different roles for the isotypes at various stages of development both during and outside of differentiation.

Kpnas have roles in many cellular processes [11,13,19,30,32]. The observation that four Kpna proteins increase their expression with differentiation while one decreases suggests that OL differentiation imposes a high demand for these import proteins and nuclear import more broadly. Indeed, successful OL differentiation requires a complex and coordinated intrinsic transcriptional program, and a diverse set of cargo is needed to enact this program [1]. In line with this, our data show that complete inactivation of canonical nuclear import strongly inhibits OL differentiation and, at higher levels of pharmacologic inhibition, results in programmed cell death, the latter also suggesting a potential role for nuclear import in maintenance of cell viability.

Interestingly, Kpnas may also play specific roles downstream of different extrinsic pro-myelinating cues. In our study, *Kpna4* appears indispensible to OL differentiation induced by CNTF, but has a much more minor impact on T3-mediated differentiation. It is quite possible that it confers CNTF-specific roles, shuttling transcription factors that play a role in Jak-Stat signaling, the major signaling pathway downstream of CNTF [33]. Indeed, Kpna4 has been shown to carry Stat3 as a cargo in other cell types, even independent of its phosphorylated (activated) state [34], and Kpna proteins also carry Klf transcription factors [35,36], such as Klf6, which we have shown to be linked to Jak-Stat signaling in CNS myelination [24]. This suggests that at least one Kpna may have gp130 pathway-specific, or Jak-Stat-specific, functions. A wider precedent also exists for Kpna-dependency of Stat proteins, although not necessarily in the context of CNTF-gp130 signaling. For example, it has been shown that Kpna1 mediates type I interferon-induced ISGF3 (Stat1/Stat2/IRF9) complex nucleocytoplasmic import [37]. In addition, Kpna1 is also implicated in Stat1 import in response to type II interferon signaling activation [38]. Other paralogs may similarly function downstream of and in coordination with specific extrinsic cues, warranting further exploration. A more useful understanding of specific Kpna paralog functions will also derive from examination of their unique cargo affinities. Although outside the scope of the current work, future studies could combine co-immunoprecipitation of the different paralogs with mass spectrometry at various stages of the OL lineage, to obtain a more complete accounting of their cargo. Such work should not only implicate the Kpna isotypes in known and essential OL differentiation mechanisms, but may also identify, in addition to novel transcription factors, regulators, and epigenetic modifiers that can play important roles in development and CNS myelination. Such findings could also potentially be extended to studies in remyelination, to improve our understanding of the mechanisms that control myelin repair and return of function in demyelinating conditions such as MS.

Examination of Kpnas in the context of the OL lineage has been a relatively under-represented area, and we have begun to examine their broader roles in OL development, particularly in differentiation. While recent work from our laboratory clearly demonstrates a role for *Kpna1* in OL differentiation and viability [24], the current study provides evidence for broader Kpna paralog involvement in OL differentiation. Studies of karyopherins may also inform development of new treatments for disease. Pharmacologic inhibitors or activators of specific isotypes, should they be developed, could have significant therapeutic value. For example, if future studies identify a specific Kpna or subtype as responsible for importing a differentiation inhibitor into the nucleus in the context of stalled remyelination in MS, a drug inhibiting its nucleocytoplasmic transport could produce beneficial therapeutic outcomes. Indeed in other contexts, such as cancer, specific nuclear import inhibitors are being studied as effective treatments [39–42]. Collectively, findings from this study implicate differential nuclear import as an important factor regulating OL differentiation. Elucidating the functions of this canonical mechanism in both physiology and disease has exciting implications for the generation of new treatments in white matter disorders.

## Materials and Methods

### Mice

Work was approved by Institutional Animal Care and Use Committee at the Icahn School of Medicine at Mount Sinai under protocol numbers LA13-00029 and LA12-00235 and adhered to the American Veterinary Medical Association guidelines. Our institution has an Animal Welfare Assurance on file with the Office for Laboratory Animal Welfare. The Assurance number is A3111-01. All mice used in this study were C57BL6 from Jackson Laboratories (Bar Harbor, Maine).

### Oligodendrocyte progenitor cell cultures

Mouse OLP were isolated as in [24]. Briefly, cerebral cortices from P6-P8 C57BL6 mice were removed, mechanically dissociated, and then chemically dissociated using papain buffer (1mM MgS0_4_, 20mM Glucose, 2mM EGTA, 2mM NaHCO_3_, 1.65nM l-cysteine, 125U/ml DNase-I (Sigma), 20U/ml Papain (Sigma), in EBSS) for 20 min at 37°C and 5% CO_2_ with occasional agitation. Chemical dissociation was terminated using high concentration ovomucoid solution (5mg/ml BSA, 5mg/ml Ovomucoid, 0.3% Glucose, 21.67mM NaHCO_3_, 104U/ml DNase-I, in 1:5 DPBS:EBSS Solution). Cells were then triturated in low concentration ovomucoid solution (1.5mg/ml BSA, 1.5mg/ml Ovomucoid, 0.324% Glucose, 23.4mM NaHCO_3_, 62.5 U/ml DNase-I, in 1:9 DPBS:EBSS Solution) until in single cell suspension. The cell suspension was subsequently centrifuged at 1200 rpm for 10 min, and resuspended in panning buffer (0.2% BSA, 5ng/ml insulin, in DPBS) and filtered through a 70μm nylon mesh, followed by sequential panning: twice on BSL1 plates for microglia depletion (15 min each), and then once on rat anti-mouse PDGFRA as primary antibody and goat anti-rat IgG as secondary (CD140a, BD Bioscience, goat anti-rat IgG) plates (45 min). Positive selection dishes were washed to remove unwanted cells, and adherent cells trypsinized and plated onto poly-D-lysine coated confocal dishes (Mat-Tek, Ashland, MA) in Sato media with the proliferative factors PDGF-AA (10 ng/ml) and bFGF (20 ng/ml). OLP were cultured in a humidified incubator at 5% CO_2_ and 37°C, with a half change of media every day. Differentiation was accomplished via the addition of T3 or CNTF at concentrations described in the Figure Legends. All growth factors were purchased from Peprotech (Rocky Hill, NJ).

### Inhibition of canonical import

The Kpnb inhibitor importazole was obtained from EMD Bioscience, and used at concentrations described in the text. Growth factors were added on top of inhibitors, maintaining described concentrations. OLP were pre-treated for 1h with concentrations of importazole indicated in the text or DMSO before the addition of T3.

### Transfection

For siRNA studies, 2μM specific or nontargeting siRNA was nucleofected into OLP via electroporation using the Amaxa Basic Primary Neuron Nucleofector Kit (Lonza Cologne GmbH, Koln, Germany), protocol O-005, according to the manufacturer's instructions. siRNA for Impα3/*Kpna4* was ON-TARGETplus mouse siRNA-Smart Pool (GE Healthcare Dharmacon, Lafayette, CO) comprising 4 different siRNA targeting regions throughout specific genes of interest. Extent and specificity of silencing were assessed by qPCR.

### Antibodies

Antibodies for immunofluorescence were as follows: CC1/APC (Mouse, 1/200, Abcam, Cambridge, MA); Cleaved caspase-3 (Rabbit, 1/6400, Cell Signaling, Danvers, MA); Ki67 (Rabbit, 1/500, Abcam, Cambridge, MA); Kpna4/QIP1 (Rat, 1/200, Santa Cruz Biotechnology, Santa Cruz, CA); MBP (Rat, 1/500, Millipore, Billerica, MA); NG2/Cspg4 (Rabbit, 1/300, Millipore, Billerica, MA); O4 (Mouse, 1/20, Dr. Peter Davies, Albert Einstein College of Medicine, Bronx, NY); Olig2 (Mouse, 1/500, rabbit, 1/500, Millipore, Billerica, MA); Secondary antibodies for immunofluorescence were conjugated to AlexaFluor 488, AlexaFluor568, AlexaFluor 647, or Pacific Blue and used at a 1/100 dilution (Life Technologies, Carlsband, CA). Antibodies for immunoblotting were as follows: Actin (Mouse, 1/1000, Santa Cruz Biotechnology, Santa Cruz, CA), Kpna1 (Rabbit, 1/1000, Proteintech, Chicago, IL), Kpna2 (Goat, 1/1000, Santa Cruz Biotechnology, Santa Cruz, CA), Kpna4 (Rabbit, 1/1000, Santa Cruz Biotechnology, Santa Cruz, CA). Secondary antibodies for immunoblotting were from Santa Cruz Biotechnologies, Santa Cruz, CA.

### Immunoblotting

Protein extracts were separated by sodium dodecyl sulfate-polacrylamide gel electrophoresis (SDS-PAGE) and transferred to onto PVDF membranes (Miliipore, Billerica, MA, USA) using a buffer continaing 25mM Tris base, pH 8.3, 192 mM glycine, 20% (vol/vol) methanol for 1h at 100 V at 4°C. Membranes were blocked for 1h in 10% milk/0.1% Tween/TBS, then incubated overnight at 4°C with primary antibodies as described in the text. After incubation in primary antibody, membranes were rinsed with 0.1% Tween/TBS three times, and subsequently incubated 2h at room temperature with secondary light-chain specific HRP-conjugated antibodies diluted 1/1000 in 10% milk/0.1% Tween/TBS. After rinsing, membrances were incubated with ECL (Thermo Scientific, Somerset, NJ) for 10 min and then revealed. For densitometry, unsaturated films were scanned using a Canon LiDE scanner (Lake Success, NY), with mean pixel density of each band measured using ImageJ software. Measurements were standardized to actin loading control, and fold change versus baseline calculated.

### Immunofluorescence

Frozen 25μm sections of paraformaldehyde-perfused CNS were immunostained. Sections were soaked in 100°C citrate buffer pH 6 for 15 min and then let to cool in the buffer to room temperature and washed three time in PBS. Samples were then incubated in blocking buffer (10% normal goat serum in PBS/Triton-X 0.3%) for 1h at room temperature, and then incubated with primary antibodies for markers as specified in the text, diluted in blocking buffer overnight at 4°C. After rinsing three times with PBS/Triton-X 0.3%, samples were incubated with appropriate AlexaFluor conjugated secondary antibody in blocking buffer for 1h at room temperature. Following rinsing, cells were mounted using DAPI Fluoromount-G or Fluoromount-G without DAPI (SouthernBiotech, Birminhgam, AL). Samples were examined using a Zeiss LSM880 confocal microscope and stacks collected using 1–2μm on the Z-axis and assembled into projections. Cells were counted in at least four stacks per condition/per marker/per timepoint in each experiment by a blinded observer using ImageJ software.

### Fractal analysis

Fractal analysis was conducted on cells expressing and immunostained for the O4 antigen which identifies immature and mature OL. Image processing and analysis was done in ImageJ software using the FracLac plugin with default settings. 15 cells per well were analyzed. For proper analysis, assessed cells were isolated without contact with other cells. Imaged cells were binarized by thresholding with default settings and then thinned using the “Skeletonize” command (a thinning algorithm within ImageJ). The box-counting fractal dimension (Db) was calculated for each cell using the FracLac plugin at default settings. This value is derived from the slope of the linear regression between the log of the number of boxes occupied by pixels and the log of these boxes’ size.

### RNA isolation and qPCR analysis

OLP lysate was prepared by the addition of 350 μl RLT solution (1ml RLT buffer, 10μl 2-Mercaptoethanol). Lysate was either used immediately or stored at -80°C for later use. RNA was isolated from cultures using a modified RNeasy Mini kit (QIAGEN) protocol with homogenization via QIAshredder (QIAGEN) and the removal of genomic DNA with RNase-free DNase (QIAGEN). RNA quality was assessed using a Nanodrop spectrophotometer (Wilmington, DE, USA). RNA was reverse transcribed in 20 μL using SuperScript RT-PCR kit (Invitrogen) and subsequently diluted to 0.5 ng/μL for qPCR. qPCR was performed using PerfeCTa SYBR Green FastMix (Quanta Biosciences) and an Applied Biosystem 7900HT Sequence Detection PCR System. The melting curve of each sample was measured to ensure product specificity. Data were normalized to *Rps11* and analyzed using the Pfaffl ΔΔCt method. Primers for qPCR are listed in **S3 Table**.

### NanoString analysis

Gene expression was quantified from isolated total RNA with NanoString nCounter Gene Expression Assay (NanoString Technologies, Seattle, WA, USA). A custom panel of murine probes was designed for target genes, normalized to housekeeping genes *Alas1*, *Ppia*, *Gapdh*, *Actb*, and *Rps11*. Following assay completion, raw data was analyzed using nSolverTM software before being subjected to statistical analysis.

### Statistics

For multiple comparisons, one-way or two-way ANOVA plus Bonferroni post-test was used as appropriate. Comparisons between two groups used Student's t-test. *p*<0.05 was considered significant. Statistics and graphs were generated using GraphPad Prism 6 software (GraphPad Software, Inc., La Jolla, CA, USA).

## Supporting Information

S1 Data Individual values used for quantitation in the text, figures, and supplementary materials. (XLSX)

S1 Table NanoString results and expression changes with differentiation. Results shown here complement data in **Figs 2 and 3**. Data provided is *Kpna* (Impα) NanoString probe count data from the OL lineage for 72h treatment with either T3 (60ng/ml) or CNTF (100ng/ml), analyzed at 24h intervals. Additional probe counts are provided for housekeeping genes and NanoString Positive and Negative controls. Transcripts were quantified from isolated total RNA using NanoString nCounter Gene Expression Assay. A commercially available panel of probes for target genes was normalized to housekeeping genes *Alas1*, *Ppia*, *Gapdh*, *Actb*, and *Rps11*. Following assay completion, raw data was analyzed using nSolver software before being subjected to statistical analysis. Expression fold change compared to an OLP baseline for each gene/treatment was derived from NanoString analysis.
(XLSX)

S2 Table Statistics for Fig 3E and 3F. Results shown here supplement data in **Fig 3E and 3F**. An ANOVA plus Bonferroni post-test was used with each gene compared within a timepoint. *p*<0.05 was considered significant.
(XLSX)

S3 Table Primer sequences for qPCR. Additional details for the Materials and Methods section. This table shows primer sequences for qPCR data presented in the manuscript.
(XLSX)
